# Putting a decommissioning programme into action: an interview study with politicians and public servants in a local healthcare organisation

**DOI:** 10.1108/JHOM-04-2023-0111

**Published:** 2024-07-19

**Authors:** Inga-Britt Gustafsson, Lars Wallin, Ulrika Winblad, Mio Fredriksson

**Affiliations:** Department of Public Health and Caring Sciences, Uppsala University, Uppsala, Sweden; Department of Health and Welfare, Dalarna University, Falun, Sweden

**Keywords:** Best practice, Decommissioning, Healthcare, Leadership, Politician, Public servant

## Abstract

**Purpose:**

A local healthcare organisation providing healthcare to 288,000 residents in Sweden struggled with a longstanding budget deficit. Several attempts to overcome the demanding financial situation have failed. A decommissioning programme was launched, and two years later, an evaluation indicated positive outcomes. The aim of this study was to explore factors politicians and public servants perceived as enablers to the successful implementation of the programme.

**Design/methodology/approach:**

A deductive content analysis approach using a framework of factors facilitating successful implementation of decommissioning decisions was applied to analyse interviews with 18 informants.

**Findings:**

Important factors were: (1) a review report contributing to the clarity of evidence, which (2) made the clarity of the rationale for change undeniable and (3) strengthened the political support for change. Additional factors were: (4) the strength of executive leadership, (5) the strength of clinical leadership supported by (6) the quality of project management and (7) a cultural and behavioural change seen as an important outcome for the path forward. A way to maximise the potential for a successful implementation of a large-scale decommissioning programme is to build a shared vision and a collaboration grounded in convincing evidence. Include public servants with a clinical background in the executive leadership team to contribute with legitimacy, competence, and trust in the decommissioning programme’s intention.

**Originality/value:**

The paper addresses the limited knowledge of best practices in decommissioning processes and contributes empirical knowledge from a successful case.

## Introduction

Local governments around Europe are facing a decrease in resources along with an increase in citizen demand on health services. This is a global challenge, and publicly financed healthcare systems are struggling with growing costs, limited resources, and citizens' increased need of healthcare (Williams *et al.*, 2017). Therefore, healthcare budget holders are increasingly forced to make decisions to decommission healthcare services (Harlock *et al.*, 2018). Decommissioning in healthcare is defined as the planned removal, reduction, and/or replacement of health care services and has been given intensified attention in the literature during the last decade (Williams *et al.*, 2017, 2021; Robert *et al.*, 2014). Several researchers have reported that decommissioning activities are complex to develop, design, and implement (Daniels *et al.*, 2013; Robinson *et al.*, 2012b). It is also pointed out that there is a desire for guidelines and advice on the best way to perform decommissioning processes (Williams *et al.*, 2017). Lessons from surveys among directors in the English National Health Service (NHS) demonstrate concerns about insufficient information and support in priority setting and decommissioning processes. Another challenge reported from the NHS is ensuring changes to be identified, accepted and implemented in the ordinary healthcare activities and that disinvestment, as well as investment, strategies ought to be developed (Robinson *et al.*, 2012a). While the concept of decommissioning originally emerged in an English context, it is pertinent to apply it to processes of change involving reductions or disinvestments in healthcare systems, including those of Nordic countries. However, these systems are typically more decentralised, with political and clinical decision-making regarding priorities and funding across various organisational levels.

### Decommissioning processes in healthcare organisations

Although decommissioning plans often are unpopular, they might allow healthcare managers to make crucial changes to reconfigure services and address economic hardship in local healthcare services (Fredriksson *et al.*, 2023). The outcomes of decommissioning processes are difficult to predict considering that the context and a multitude of factors could influence the results. Decommissioning could be challenged due to political vulnerability of the organisation, formal levels of power, and organisational self-interest (Williams *et al.*, 2021; Fredriksson *et al.*, 2023). The risk of failing when implementing a decommissioning programme is higher compared to other changes carried out in healthcare. Furthermore, there are many different types of priority setting and decommissioning processes depending on the purpose, scale, and level of the organisation that needs to carry out decommissioning decisions (Robinson *et al.*, 2012a). The factors considered valuable and important to succeed with decommissioning activities may also differ between decommissioning cases. For example, interviews with clinic managers in Sweden have shed light on some relational skills perceived to be crucial to succeed with the decommissioning process, e.g. attention to human aspects of change and clinic managers being prepared to handle tensions and strong emotions at their clinics (Gustafsson *et al.*, 2021). By contrast, the unfolding of three decommissioning cases in the English NHS showed that relational and interpersonal skills appeared to be of secondary importance to the outcome. The decommissioning process was successfully carried out in just one of the cases; in the other cases, the decisions were rejected and undermined by the stakeholders involved at the hospitals. However, the successful case was small in scale and described to be more of a managerial process than a complex change process incorporating relational challenges on many levels (Williams *et al.*, 2021). Not surprisingly, it is reported that large scale decommissioning programmes—such as the one studied in this article—are more difficult to handle and succeed with than decommissioning decisions that include single interventions (Daniels *et al.*, 2013; Robinson *et al.*, 2012a).

Decommissioning activities, as well as priority setting, is not just a technical task carried out by public servants; it requires social skills and loyalty to the process, and this could be achieved by including both politicians and the medical professionals early in the process. To have politicians involved at detailed levels in priority setting could be beneficial if it is carefully sorted out under what circumstances and in what decisions their presence is useful. Their presence and engagement in priority issues could also prevent electoral punishment and critique from the medical profession (Garpenby and Nedlund, 2016). Through a metaphor, researchers strive to illustrate the complex dynamic between politicians and public servants taking place in decision-making (e.g. in decommissioning processes): the purple zone, a blurred zone of two worlds, the political “red zone” and the administrative “blue zone” (i.e. there is no sharp line between politics and administration, instead the public servants work in an intermediate space). Public servants guide and support politicians in decision-making processes by ensuring access to comprehensible information and by using their knowledge and administrative skills in an interactive working relationship (Alford *et al.*, 2017).

In Robert *et al.* (2014) reported on Delphi rounds with experts identifying problems and barriers in decommissioning processes, such as a disconnection between technical and political aspects of decommissioning, a messiness and randomness in the form of political conviction, searches for quick fixes, and a lack of capacity to base the decisions on evidence for a longer-term sustainability. These researchers developed a framework with three categories suggesting the best practice to decommission healthcare services: change management and implementation, evidence and information, and relationships and political dimensions (4). Drawing on this framework, in this paper, we report on politicians’ and public servants’ experiences of successfully implementing a large-scale decommissioning programme in a local healthcare organisation. In view of the complex processes that take place during decommissioning, and in particular at the highest decision-making level (among politicians and public servants), not much is known about the experiences of those involved as decision-makers (Harlock *et al.*, 2018). Thus, it is crucial to empirically deepen the picture by investigating what in fact happens at the highest level of decision-making during decommissioning processes. By identifying factors politicians and public servants consider most important for facilitating decommissioning processes, this study contributes knowledge on how to increase the ability to achieve the intent of decommissioning processes efficiently and successfully.

## Methods

This study relies on a qualitative design with interviews analysed through a deductive content analysis, followed by a complementary inductive analysis.

### Setting

The healthcare services in Sweden are largely decentralised, and 21 regions have independent budgets and are responsible for planning, funding, and providing services to their inhabitants and deciding on tax rates, patient fees, and decommissioning activities. All Swedish regions are politically governed, and elections are held every four years. Region Dalarna is the size of Belgium but with only 288,000 inhabitants and healthcare services are provided at 30 primary care centres and six hospitals.

In 2015, Region Dalarna started a comprehensive decommissioning process due to a longstanding budget deficit. The region had to save about 70 million EUR between 2015 and 2019, and the savings had to be rapid since the economic situation was critical. For example, the region had to borrow money to cover the expenses for salaries and had high costs and low productivity. The region's executive leadership, consisting of politicians and public servants, developed a decommissioning programme in collaboration with clinic managers, with plans including, e.g. concentration of some services to more urban areas, efficiency improvements, changed staffing policies, and the closing down of a rehabilitation centre and a local ambulance station. Before starting the decommissioning programme work, regional public servants compiled a review-report on the region's situation, that was used at all levels in the organisation, which included information such as demographics, quality of care in each speciality, costs and staffing levels (Dahlström *et al.*, 2015) and Appendix 1. The decommissioning programme intentions were approved by the ruling coalition in the Region assembly and supported by one of the opposition parties (Laggar, 2015).

The overall evaluation criteria were that the changes established in the decommissioning programme should improve the region's economy without threatening patient safety and quality of care. By the end of 2016, nearly 150 (almost 95%) of the decommissioning activities decided in 2015 had been implemented, and a positive trend in the region's economy was reported. In 2020, a national evaluation reported that Region Dalarna had the lowest healthcare cost per inhabitant among Sweden’s 21 regions (KOLADA, 2020). Furthermore, there was, unexpectedly, little resistance from the clinics and clinic managers involved in the decommissioning activities in relation to the many and extensive changes they had made (KPMG, 2016; KPMG, 2018; Fredriksson and Moberg, 2018). A national evaluation indicated a positive trend in quality of care for the primary care, surgery, and medicine divisions during the decommissioning processes, as well as in indicators of patient safety (Yang and Buijs, 2019). Finally, an employee survey showed improved results regarding work motivation and the perception of the region's leadership (SALAR, 2019). In conclusion, most evaluations showed that the implementation of the decommissioning programme progressed positively, which indicated that the decommissioning processes were successful.

### Sample

Local health systems in Sweden, so called regions, often have a leadership team headed by political county commissioners and non-political public servants. Eighteen individuals from the leadership team in Region Dalarna were invited to participate in the study, (Table 1). The selection of the informants was based on the informants being assigned to and highly involved in developing and implementing the decommissioning programme. They were invited by e-mail and received information about the study's purpose and that participation was voluntary. All the invited 18 individuals decided to participate. At the time of the interview, they were verbally informed about the study, including the option to withdraw at any time. Prior to the interview, the politicians and public servants gave their written consent to participate. The study was approved by the regional ethics board in Uppsala (No. 2016/504).

### Data collection

The interviews were carried out by the first and last author. A semi-structured interview guide was developed by the research team, based on theories of welfare state retrenchment, change management, and implementation literature (Starke, 2006; Damschroder *et al.*, 2009; Kotter, 1995) and Appendix 2. The questions were designed to capture how the informants experienced the decision-making process leading to the restructuring plans and how responsibility, interaction, and legitimacy issues were handled throughout the decommissioning process the first two years of the four-year decommissioning programme. Furthermore, the politicians and public servants were asked questions about their own role and efforts in implementing the decommissioning programme. The interviews lasted between 45 min and 1 h, were audiotaped with permission, and conducted at the politicians' and public servants' workplace in the region's administrative building between January 18 and April 3, 2017.

### The framework

The framework used is constructed on factors considered to shape successful implementation of decommissioning decisions (Robert *et al.*, 2014). Thirty international experts participated in the development of the framework and, through Delphi rounds, contributed with views on what should shape decommissioning processes. The experts agreed on three factors that ought to inform decommissioning processes: quality and patient safety, clinical effectiveness, and cost effectiveness. The experts also pointed out factors that they perceived—in practice—informing decisions to carry out decommissioning, such as cost/budgetary pressures and government intervention. They concluded that the best practice in decommissioning should be split into three factors: change management and implementation, evidence and information, and relationships and political dimensions. These three factors and 30 underlying subcategories were used as the analytical framework in the study (Table 2).

### Analysis procedure

The analysis was carried out in three steps, with the first step being a deductive content analysis (Elo *et al.*, 2014; Polit DF, 2012). First, the interviews were fully transcribed verbatim, and the interviews were read several times by the first and last author to get a perception of the entire interview material. The first author used NVivo 11.0 in assisting the coding of relevant interview material into categories and subcategories matching the factors in the framework by Robert *et al.* (2014) (Table 2). The coded material remained as quotes in the subcategories throughout the analysis procedure. The last author read the subcategories and verified the findings to secure reproducibility (Krippendorff, 2013). A small number of interpretations differed, and the two authors re-read these sections and discussed to reach precision and consensus. All authors read and discussed the coding of three interviews to ensure dependability and confirmability. The result of the deductive content analysis is presented in Appendix 3.

**Figure 1:**
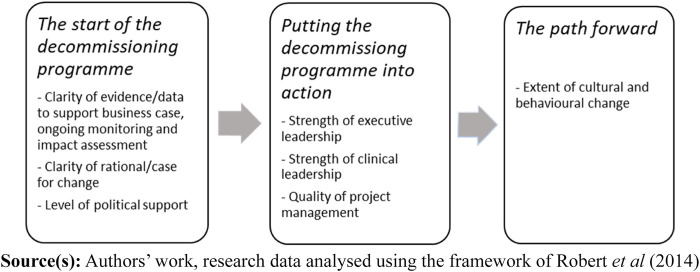
Timeline illustrating the seven key factors identified as facilitating distinct parts of the process in the decommissioning programme

The subcategories considered most crucial for success in the decommissioning process (mentioned and highlighted the most by the respondents) underwent a second analysis. These subcategories (seven key factors) were placed in a timeline to illustrate when factors explicitly facilitated distinct parts of the decommissioning process and contributed to a successful outcome according to the informants (Figure 1). The timeline is also used as a structure for the result section.

In the final step, the parts of the interviews that related to the seven key factors were re-examined by two authors that identified and discussed a frequent and specific theme characterising these factors. By inductively identifying this common characteristic, an important overarching theme of the facilitating key factors was highlighted. The presence of this theme was pointed out by the informants as decisive in their efforts to plan, implement, and maintain the intent of the decommissioning programme. Selected quotes are integrated in the results to illustrate and give colour to our findings.

## Findings

We identified seven key factors (subcategories) the politicians and public servants perceived to be the most important in enabling the decommissioning process. The results are presented as a narrative of the decommissioning process illustrated as a timeline (Figure 1). Initially, *“the start of the decommissioning programme”* consists of three subcategories as well as the second part, *“putting the decommissioning program into action”.* The last part, *“the path forward”,* includes the facilitating factor of cultural and behavioural change. Finally, a presentation of the theme trust, identified to be strongly related to our findings, is presented. Quotes are numbered 1 to 18 representing the informants.

### The start of the decommissioning programme

The informants described that after 19 years of budgetary deficits, the executive leadership in Region Dalarna decided that a detailed action plan should be drawn up to address the region's economic problems. In connection with this, a comprehensive review-report covering data about costs, staffing levels, quality of care, and demographics was presented. Compared to other regions in Sweden, it was found that Region Dalarna had a significantly higher cost level. A public servant expressed his/her worries at the time: “*We had the worst economic situation in the whole country. We were forced to borrow [money] to pay salaries. So, it is quite clear that we were then at the bottom and going even further down. (…) it was a, how can I put it ….an emergency situation. We had to do something.”* (Informant 18).

The executive leadership team was convinced by the data presented in the review-report and mentioned that, at that point, the “clarity of evidence” was no longer deniable and that the region was in an extremely difficult situation. “*The review-report was somehow the big “breakthrough”. (…) it became very clear that there were several obstacles along the way, but basically, once the review-report had been compiled, it was difficult to question its content.”* (Informant 8). Managers, at all levels in the region, were now required to commit, involve, and prepare themselves and their employees to work hard to solve the difficult and escalating financial situation. A public servant emphasised that a “rationale for change” eventually was adopted and that this might be the opportunity to make sustainable changes: “*It was a very transparent and understandable presentation of the situation. We could not go on like this. We were presented a relevant description of what the actual situation was in Region Dalarna. We took it to our hearts and understood that we had to do things differently in the future.”* (Informant 8).

The interviews illustrated that the politicians realised that the situation was very serious and that they were prepared to make difficult decisions and, if necessary, reduce, reallocate, and even close down healthcare services to ensure an improvement of the region's economy. A division manager claimed: *“The fact that it was so bad made it easier for us to convey a situation of a crisis and get a common understanding, even internally, that we needed to take action.”* (Informant 5).

The new approach among the politicians facilitated and justified the decommissioning decisions. A public servant described what he called the new “level of political support*”*: “*I am glad that the political leadership, in my opinion, has taken a huge responsibility in this project and has been prepared to make tough decisions. Regardless of party colour, I think there are many good examples of politicians being prepared to think new and creative.”* (Informant 3).

The “level of political support”, joint responsibility, and ability to find common solutions between politicians and public servants were perceived as important factors in the success of the decommissioning process. An experienced public servant expressed his/her respect to the politicians: “*Thanks to the fact that politicians had been involved, from the beginning, they knew how to refine the decommissioning proposals, e.g. which words to use. That made it go smoother than I thought it would.”* (Informant 2). Another public servant stated: *“Politicians are more knowledgeable than we. They can better see what may upset the public.”* (Informant 7). Relational skills and the capability to negotiate, give, and take were invaluable qualities when difficult issues were discussed. Confidence in decision-making processes and perceived strong cohesion in the executive leadership team were also reported as important values in the collaboration between politicians and public servants.

### Putting the decommissioning programme into action

According to the informants, the region's executive leadership had been under criticism for many years, and an inefficient leadership had been pointed out as one reason for the region’s critical economic situation. The fact that the region had repeatedly failed to address the financial situation confirmed the picture, and it became obvious that the region was facing a crisis and needed to act promptly, take the lead, and establish a “strong executive leadership” to handle the situation. The responsibility and loyalty to the decisions made by the executive leadership team before the implementation of the decommissioning programme was described by some as non-existent. Several statements in the interviews described clinic managers that basically ignored decisions on cutbacks, and some informants called this behaviour annoying and unfair.

The executive leadership team was also aware of the demands and challenges of leading highly qualified professionals and described the situation like this: “*Yes* *…* *there are many clinic managers who would make it easy for themselves and seek popularity by agreeing with their employees and saying, “Look at the executive leadership team, they do not understand a thing”*. *How easy isn’t it to do that as a clinic manager when you feel a lot of pressure from strong professional employees …. To guide, support and lead an organisation with highly qualified professionals, is truly very demanding for a clinic manager.”* (Informant 7). The awareness of these challenges made the executive leadership team realise that they had to pay attention to the “strength of clinical leadership”. In the interviews, it was mentioned that considerable efforts were made to strengthen the region's leadership at all levels throughout the decommissioning process. A new leadership program was implemented, and a major theme in the educational program was the clarification of roles and responsibilities as a first line and clinic manager.

Leadership issues were also constantly addressed in joint discussions at all levels in the organisation. An important theme on the agenda pointed to the common responsibility and that all clinics must help to overcome the region's difficult economic situation. At this time, many initiatives were taken to develop the “strength of clinical leadership”, and one informant said: ” *I expect from clinic managers that they should be able to see the total picture and understand their part in that picture and what role to play. I think there has been a better understanding among clinic managers of their roles during the last couple of years …... We had these big meetings, talked about emotions, talked leadership, talked about what a manager's task is.”* (Informant 7).

The informants illustrated that the executive leadership team included persons with experience from clinical leadership and change management and that their skills were appreciated and strengthened the “quality of project management” in the decommissioning processes. The importance of involving and genuinely ensuring engagement at all levels of the organisation was a crucial success factor according to an informant with lived experience from leadership and change management: “*What I call “mobilisation” is to involve all forces within an organisation when facing a challenge. Because it is then you establish the necessity for change, while you at the same time get good ideas. So, I was incredibly happy that we had come so far in our organisation, so that we could affirm this approach.”* (Informant 1).

The fact that the region’s economic difficulties were communicated in the media, to all clinics, to all unions, and continuously updated and presented on the region's internal and external web pages every week, made it difficult to deny that information about the decommissioning programme was missing. A division manager thought this was proof of “quality of project management: “*We were careful to include the union representatives from the first moment and I think that it contributed quite significantly to the anchoring process among the staff and gave it additional legitimacy.”* (Informant 18). In this way, all shared the narrative and realised that it was an end to the waste of resources and that they now had to merge forces and focus on the new path forward. An experienced public servant considered it important to avoid creating a bad atmosphere by accusing a particular clinic manager of being wasteful and described an alternative strategy: “*We made it a collective problem. We have many systemic deficits that everyone needs help to straighten out. It was probably also good because then you avoid people trying to find scapegoats in all this. We took it as a collective problem that the region has a potential for improvement here.”* (Informant 8).

A tricky question in the executive leadership team was to establish and attain the “quality of project management” to implement the decommissioning programme. The executive leadership realised that this was a truly demanding task and that it required competence, structure, and tenacity to execute the decommissioning plans. In the past, the region had engaged external consultants to run large projects, and it was mentioned in the interviews that this was discussed this time as well. However, the executive leadership team changed their mind and stated that clinic managers and healthcare professionals had to be given responsibility for both the process and outcome of the decommissioning program this time. One public servant explained: “*We have previously been working with consultants. They are good, but when they have finished their work, they take the knowledge with them as well. There are also negative attitudes towards external consultants. “The consultant did not understand anything”, “It is just shit what they suggest”. Some think they are worth gold, but they still do not want to implement changes because they do not feel any real responsibility.”* (Informant 1).

By being handed a considerable part of the responsibility for both the process and outcome, the clinic managers became involved in the process early and contributed to a large extent with savings proposals, and valued patient-safety risks related to the proposals. The four newly established divisions were described as well functioning groups, with clinic managers and a division manager heading the division, “strengthening both executive and clinical leadership”. In the divisions, a lot of discussion took place, i.e. possible decommissioning decisions were raised, priority issues framed, and ethical discussions took place. According to many informants, frequent meetings in the divisions were invaluable in the decommissioning processes and, according to one division manager: “ *…. there were, of course, many tough decisions to be made to identify potential cost-cutting activities in a dialogue with the clinic managers and with my colleagues in the executive leadership team and at the same time ensure a high level of involvement and commitment from our different clinics* *… It was not always that we could come to an agreement, but still the importance is that the idea was presented.”* (Informant 3).

The executive leadership at this time was more stable, knowledgeable, and determined than compared with previous attempts to improve the economy, which was perceived as an important factor several informants pointed out to succeed in the implementation of the decommissioning program. It was, quite often, under intense pressure from the medical profession, opposition, and the citizens. Some decisions caused irritation and protests, and the executive leadership team was repeatedly exposed to harsh criticism in press and media. One respondent recalled a situation that portrayed “strength of executive leadership”: “*So (a division manager) has now been out and been heavily criticized and yelled at* *… but he is insistent and will not change his mind. I think that is great …... Even if you feel that the situation is no fun, it actually could give you a bit of legitimacy. It is important to show that you will not give in. I think that this is also a success factor.”* (Informant 10).

### The new path forward

The interviews highlighted that after several previous attempts and shortcomings to overcome the region's demanding financial situation, the employees in Region Dalarna were tired, and many felt resignation about new versions of cost-cutting programmes. Earlier experience with general cost-cutting programs by lowering cost levels at all units by the same percentage had failed. Some of the clinic managers had tried to reduce costs at their clinics, but many felt that their efforts did not make any sustainable improvement of the economic situation, either at the clinic or for the region. A division manager referred to a clinic manager that had asserted: “*So, the goal was to save and, honestly, that is never fun. But some have the attitude that: Well, we have been through this before. It's just a matter of crouching a little and then it will pass.”* (Informant 2).

To increase cost awareness, discussions were held at the clinics about new expensive therapies and difficulties in keeping costs at stipulated levels. According to some of the informants, these discussions about the clinic’s scope and content of services contributed to revising the services at many clinics; several treatments were reduced, and some patients were referred to private healthcare providers. One politician implied how he/she felt these issues had been dealt with in the past: “*We have not dared to approach the real issue here in Sweden at all; What should Swedish healthcare, tax-financed, offer the Swedish population? We can't do everything and from there on keep adding things on top of everything.”* (Informant 10).

As a first step to cut costs, the division and clinic managers sought to reduce or stop providing non-essential care at their clinics. This required, in some cases, brave political decisions to support the decommissioning decisions to be carried out. In some issues, these decisions resulted in harsh protests from citizens regarding services that were completely shut down, e.g. training and rehabilitation pools, and satellite primary care centres in rural areas. Despite the tough criticism, nearly all decisions in the restructuring plans were implemented. The decisions were often endorsed by the fact that they were necessary to be able to provide the population with high-quality care in the future. These discussions about the content of care in specialised care and at primary care centres would become a recurring and long-awaited topic of discussion at clinics and, in that way, a “cultural and behavioural change”.

This important “cultural and behavioural change” influenced managers in the region, during the implementation of the decommissioning programme. The managers developed and broadened their views, from their own narrow task to an overall responsibility of the region’s economic hardship. During discussions, the division, clinic, and first line managers’ responsibility was clarified and inescapable, and for some, the sometimes-crippling feeling that it is always someone else's fault faded away. “*There is a process from starting to think; Oh my God* *… do we really need to do this? It's the politicians' fault!* *…* *To realize that; No, it is I who must take responsibility for making sure that it works at my clinic, that is my responsibility, it is not the politicians' responsibility that things do not work at my clinic. I am the one actually paid to make it work.”* (Informant 10).

### Trust

In addition to the deductive analysis, the overarching theme “Trust” emerged in the analysis. “Trust*”* was a recurring subject in the interviews and, particularly, in the narratives related to the seven key factors that facilitated the decommissioning process. In the beginning, the review-report was mentioned as invaluable to establish trust in the decommissioning programme. A politician explained his/her feeling: “*As we are not representatives of the profession, we must be able to trust that we get evidence-based material with a reliable analysis that we can rely on when making decisions.”* (Informant 15).

A feeling highlighted by many informants was the importance of trust in each other, no matter the difficultly of tasks they were forced to handle. An experienced politician reflected on his/her role: “*And there must be a trust as well. Because what has happened in many regions, when the criticism has started, is that the politicians have given up. Region directors have been replaced; I do not think this will solve anything. But I think it has been important that I have been visible … …. that they can trust me and that I will never let them down, that I will never abandon them, I stand behind them whatever happens. I have become very well informed [by the public servants]. I think that is important, that I was able to have a really good dialogue with them.”* (Informant 10).

When the decommissioning programme was put into action, the trust in a competent, knowledgeable, and strengthened executive leadership was perceived as vital by the clinic managers, according to discussions in the divisions. The division managers (four public servants with a clinical background) led the divisions and managed to establish trust and legitimacy among the clinic managers to engage and be loyal to the hard work of carrying through the changes in the organisation. On the other hand, the decision to hand over the responsibility to the staff to identify possible decommissioning proposals was another way of showing trust in the professionals’ ability to guide the process. When clinic managers had an awful lot of red numbers in their economic results, a public servant demonstrated his/her trust in their capability by this declaration: “*To be able to act in a supporting and not panicking way is, however, very important so that the clinic managers feel strong and inspired to resolve things even when they are demanding.”* (Informant 7).

An increasing awareness of the necessity of a cultural and behavioural change as the only path forward also indicates trust among employees to the intent of the decommissioning programme, regardless of problems along the way. During the sometimes-demanding change process, a politician pointed to the trust in receiving support in difficult situations: “*You know where you are going, you know that you are backed up all the way from your manager to the politicians. It is necessary for you to have that feeling of being secured, supported because you could get a knife in your back when changes become too difficult for somebody.”* (Informant 10).

## Discussion

This study provides an empirical contribution to the field of decommissioning policy and practice by unfolding a successful case of developing and implementing a decommissioning programme. The process is viewed through politicians” and public servants” lenses, describing their experiences and efforts in implementing a large-scale decommissioning programme. In interviews, politicians and public servants pointed to seven crucial factors that facilitated the successful implementation. These seven factors were summarised in a three-part chronology. First, at the start of the process, a solid review-report contributed evidence that made the rationale for change undeniable, and this strengthened the political support for change. Second, strengthened leadership capability both in executive and clinical leadership teams, that were given the responsibility to lead the entire decision-making and implementation of the decommissioning programme, turned out to be a success factor. Third, a cultural and behavioural change among managers and employees, towards an acceptance for a more responsible use of resources, was considered the most valuable outcome and the path forward. Furthermore, trust was considered as an overarching attribute to all the seven key factors.

Understanding the multifaceted relationship between politicians and public servants can help reveal the difficulties that may affect the decision-making of decommissioning programs. Among other things, power and the availability of information and personal relationships are at stake in local healthcare decision-making processes (Joensuu and Niiranen, 2018). The experiences reported in our study infer that these potential difficulties had been adequately addressed. Scholars describe that policy is created by street-level bureaucrats, at the bottom of the organisational hierarchy (Lipsky, 2010). In this case, policies (decision-making) were created at many different levels in the organisation. Public servants (clinical) offered flexible support, decision-making by moving between and connecting different stakeholders and levels of the organisation. The review-report covering information about the region's economic situation, staffing, and quality of care was available to both politicians and public servants and served as a stable start to discuss the escalating critical economic situation and the rationale for change. This information contributed to a fruitful and equal discussion between the two groups of stakeholders trying to figure out how best to deal with the crisis. Similar experiences have been reported when national clinical guidelines were implemented in Swedish healthcare contributing to a more constructive dialogue between politicians and public servants in order to achieve a more equal healthcare system (Sandberg *et al.*, 2019). With this in mind, blame-sharing could be a way to understand the dialogues often taking place between politicians and public servants when decommissioning proposals are discussed, justified, and decided in healthcare organisations (Fredriksson *et al.*, 2019). For example, the withdrawal of seven satellite primary care centres in rural areas resulted in harsh public protests but politicians and public servants jointly defended the action. These healthcare services constituted ”a sense of belonging, and identity of the local community” (Kvåle and Torjesen, 2021), that despite public protests was carried out due to unity among the politicians and public servants. The large number of decommissioning decisions made it difficult for patients and citizens to keep track of all changes, which were also carried out with high pace. Although there were some loud protests from particularly exposed groups (e.g. from the disability movement), an evaluation showed that the public overall were aware of the decommissioning programme and surprisingly, that the trust in the regions' healthcare system increased during this time, most notably among the elderly (Fredriksson and Moberg, 2020). Patients and citizens were not involved in a structured way in establishing the decommissioning programme. Public involvement may have enriched the decision-making processes, but it would also have risked making the processes lengthy, complex, and difficult to carry out with acceptable pace and quality (Connelly, 2005).

This time the executive leadership decided to use a new approach, as previous cost-cutting programmes lowering cost levels at all units by the same percentage, had failed. The findings that an early involvement of clinical leaders in decommissioning processes is a critical success factor, as well as the need of a strong executive leadership team to handle, coordinate, and support decommissioning activities, is supported by several studies (Daniels *et al.*, 2013; Robert *et al.*, 2014; Gustafsson *et al.*, 2021) and in line with how the present programme unfolded. Furthermore, to achieve intended implementation outcomes, it is also necessary with a tight, thorough dialogue between the executive leadership and the clinic managers accountable for the implementation of the changes (Uvhagen *et al.*, 2018). In the current study, the important dialogues to grasp the clinic managers' perceptions of the decommissioning process often took place in the newly established divisions. In these recurring meetings, issues about ethical considerations, difficulties, or opportunities linked to the responsibility to execute the decisions were discussed. The executive leadership team was at this time strengthened with public servants with a clinical background (four division managers). This made the dialogue and decision-making process smoother, and issues could be referred to the division manager, clinic manager, or first line manager perceived as the most competent to solve the specific problem. This flexibility increased the ability to involve healthcare professionals best suited to propose changes and to make wise decisions. Similarly, results from a previous study reported a higher success rate when healthcare professionals had the opportunity to influence changes in healthcare organisations by being more involved in the decision-making (Nilsen *et al.*, 2020). To some extent, this flexible leadership approach detected in our study is in line with previous research that points out that healthcare leaders need to be flexible, reflective, and swiftly adapt new approaches that better suit the actual situation and context (Clarke *et al.*, 2021).

The new leadership program in Region Dalarna, in which all managers were invited to participate, was highlighted for having played an important role by strengthening participating managers' leadership skills and was mentioned as a facilitating factor to a successful outcome of the decommissioning program. In line with this, researchers report that employees describe their managers as more confident, flexible, and observe their managers as more eager to change their behaviour after the two-year leadership development program (Palm *et al.*, 2015).

The executive leadership worked hard to establish a common sense of responsibility among clinic managers and presented the economic hardship of Region Dalarna as a collective problem. This may have facilitated the change process by broadening the managers' view and contributed to engagement and responsibility. By contrast, it is demonstrated that clinic managers could also act out as competitors and representatives for their own clinics or hospital (Edmonstone, 2009), which was not perceived as a major problem in the present study as the newly established divisions facilitated and encouraged peer support.

A recurring finding in the current study is the importance of trust that seemed to be highly valued in all parts of the decommissioning process. Being able to trust each other was described by many informants as mutually crucial for both leaders and those who were led in their efforts to implement the decommissioning program. The executive leadership team in Region Dalarna, strengthened by public servants with a clinical background formed a robust, reliable, and competent core of the leadership that succeeded to create trust. Decades of diverse research in sociology, psychiatry, and management make it clear that trust has become an important research topic, like a multidimensional concept, that seems to play an important role in organisations' opportunities of succeeding and functioning effectively (Luhmann, 1988; Rousseau *et al.*, 1998; Lewicki *et al.*, 2006). The literature refers to different forms and levels of trust between individuals, within teams, and in organisations (Fulmer and Gelfand, 2012). Vanhala *et al.* (2016) emphasise that an impersonal dimension of organisational trust (impersonal trust) occurs when employees set their trust in their management team as a unit and functional structure in the organisations as an alternative to relying on a specific manager or decision-maker (interpersonal trust). The results also demonstrate that impersonal trust increases commitment and enhances trust when employees experience positive behaviour from the organisation (Vanhala *et al.*, 2016). This is consistent with our findings, where the informants, to a large extent, illustrated being encouraged and supported by colleagues in a unified executive leadership team.

The fact that financial pressure constantly is on the agenda in publicly financed healthcare organisations makes this study relevant to many other healthcare systems and settings. There is a need to make decommissioning decisions in almost all healthcare systems, which involves tough choices, difficult conversations, and decision-making processes about priority setting to be dealt with. This study illustrates the often-messy nature of decommissioning processes, highlights the important relationship between facilitating factors and trust, and provides an account of how challenges were handled along the way. The main contribution of our study is its empirical base and provision of useful knowledge from a successful large-scale decommissioning process. From this real-world decommissioning programme, factors identified as crucial could help healthcare organisations to achieve their decommissioning decisions. Even though a multiflora of institutional arrangements and reimbursement systems exist, the seven import factors identified in this case ought to be universal and could facilitate change processes initiated by resource scarcity. Carefully planning, designing, and creating optimal conditions, considering the most important facilitating factors, may reduce the risk of failing, wasting time, and losing employees' trust when implementing decommissioning decisions. That being said, some specific conditions likely contributed to the successful implementation: clear crisis awareness developed over years of economic challenges, which underscored the necessity for radical changes, as well as long-standing relationships between politicians and public servants in the relatively small-sized region.

The implementation of the studied decommissioning program was considered successful. Although success in this case means that the restructuring plans were successfully implemented and evaluations showed positive results, there may be decisions that affected some patient groups more negatively, for instance, patients living in rural areas who lost their healthcare centre and rehabilitation clinic. Thus, it is important to further investigate adverse effects of decommissioning programs. Another limitation is the risk that informants wanted to portray the decommissioning process in a too successful way. To monitor this problem, politicians from the opposition were among the informants and their experiences did not diverge substantially from those of the politicians responsible for the implementation of the programme.

## Conclusions

In summary, to increase the ability to implement a large-scale decommissioning programme efficiently and successfully, there is a need to consider and ensure the presence of fundamental facilitating factors adapted to the specific context of the organisation. In this study, seven factors perceived to be crucial in implementing the changes in a credible way, each in different parts of the decommissioning process. In the complex processes of decommissioning, public servants and healthcare professionals require stable evidence making the rationale for change convincing to justify necessary changes and establish political support. Furthermore, a key factor when putting the changes into action is a strong executive leadership supported by public servants with a clinical background to develop trust, technically and in relationships in all parts of the process, as well as in the intent of the decommissioning programme. Other key factors are to early involve and give clinical leaders and healthcare professionals both opportunities and responsibility to participate in the decommissioning processes. To prepare for future demands resulting from economic hardship or other threats to healthcare organisations it is essential to preserve and refine the experiences and knowledge achieved during successful implementation of decommissioning decisions in healthcare organisations.

## Figures and Tables

**Table 1:** Characteristics of the informants

Region politicians	Public servants (non-clinical)	Public servants (clinical)
• Region commissioner and chairman of the executive board, Social Democrat (until October 1, 2016) (f) • Region commissioner and chairman of the executive board, Social Democrat (after October 1, 2016) (m) • Region commissioner (Left party) (f) • Leader of Centre party (opposition) (f) • Leader of Liberals (opposition) (m) • Leader of Christian Democrats (opposition) (f)	• Region director *(f)* • Deputy region director *(m)* • Health-care director *(f)* • Chief financial officer *(m)* • Chief analyst *(m)* • Development strategist *(f)* • Head of patient choice office *(f)* • Chief medical adviser *(m)*	• Division manager: medicine (m) • Division manager: primary care (m) • Division manager: psychiatry (m) • Division manager: surgery (m)

**Note(s):** Informants *n = *18; gender: female (f) *n* = 8, male (m) *n* = 10

**Source(s):** Authors' own

**Table 2:** Framework by Robert *et al.* (2014) rating factors in descending order, in terms of importance within each category, shaping the extent to which decommissioning is implemented as planned

Factor
*Change management and implementation strategy*
• Strength of executive leadership
• Strength of clinical leadership
• Quality of communication
• Clarity of specific aims and objectives at start
• Extent of cultural and behavioural change
• Attention throughout to human aspects of process of change
• Quality of project management
• Availability of resources to support decision-making and implementation processes
• Quality of strategic planning
• Training and preparation of staff
• Clarity of incentives and levers to support change
• Complexity of decommissioning programme
• Pace of change
*Evidence and information*
• Demonstrable benefits
• Clarity of evidence/data to support business case, ongoing monitoring and impact assessment
• Clarity around new patient pathways
• Review/evaluation of process
• Availability of alternative services
• Extent of adoption elsewhere of new intervention/service
*Relationships and political dimensions*
• Clarity of rationale/case for change
• Nature and extent of clinician engagement/involvement
• Level of political support
• Transparency of decision-making process
• Nature and extent of patient/public engagement/involvement
• Quality of partnership working with relevant agencies
• Extent to which challenges vested interests
• Nature and extent of media coverage
• Stability within the local health economy during transition
• Reputation of existing providers
• Meets community expectations

**Source(s):** Robert *et al.* (2014)

## Appendix 1 Table of contents, review-report. Dialogue material for clinic managers

### Evaluation of a health care system

Prerequisites for comparisons in the field of economics
Areas
Comparison county councils

### Economic sustainability

Costs
Exemplification of the model
Efficiency

### Economic sustainability – results

Total cost base
  Cost by area
Input factors
  Drug
  Purchased care
Buildings
Staff

### Quality

Quality and costs
Open comparisons
What might be important to measure?
Index calculation
Overall results
Costs of healthcare-associated infections, pressure ulcers and healthcare injuries
Patient-reported measures
Secondary preventive measures

### Staff

Hired staff
Retirements
Staffing
Sick leave

### Population of the county council

Age
Education
Foreign-born persons
Unemployment
  Public health developments
Analysis of the health situation in Dalarna “Health on equal terms 2014″
Self-rated health
Impaired mental well-being
Risk use of alcohol
Smoking habits
Obesity
Pain

### Availability

#### Structure:

Based on cost base:
Based on physical supply points:
Primary care
Based on the number of providers who perform each procedure/treatment:
References

### Capacity

#### Strategy and goal follow-up

Analysis
  Strategic Maturity Assessment
  BI Maturity Assessment (BIMM)
Conclusions
Next steps

### Revenue

**Source(s):** Dahlström *et al.* (2015). Vägen till ett välvårdat Dalarna

## Appendix 2 Interview guide

### Background notes

#### Profession

What is/has been your role in the work with the implementation of the decommissioning programme?

### Change management: problem definition

1. How was the problem facing Dalarna defined? Who was involved in formulating the problem definition? What “evidence” was presented to strengthen the problem definition?
2. What was considered the cause of the problems? Did any actor have a different view? Do you agree?

### The change process: motives, goals and content

1. In your opinion, what were the main reasons why the decommissioning programme came into being?
2. What do you perceive as the most important content of the decommissioning programme? What was the goal of the changes announced in the decommissioning programme? Do you perceive the goals as relevant?

### Change management: problem solving

1. How was the solution to the problems Dalarna County council faced (overall) formulated? What did you think needed to be done? Who was involved in formulating the solution? Who was the driving force in that process, according to you?
2. Did you feel that there was agreement or disagreement about what the solutions should be?
3. How did they choose in which areas reductions or priorities would take place? Were there any underlying principles for the choices? Geography, healthcare topics etc.? How did this particular part of the process happen? Negotiations?
4. What was at stake if changes were not decided? Do you think you had the time to do the necessary analyses of the potential effects of the changes that were decided?

### Change management: responsibility and legitimacy

1. Who do you feel took responsibility for the announced cuts to the residents and patients of Dalarna? Who took responsibility towards the staff? How did responsible politicians and officials justify the cuts?
2. How do you feel the county council worked to create legitimacy within the organization for the cuts that needed to be made? Do you think you succeeded? If not, why not? Was there anyone or some that you think were central in creating legitimacy within the organization?
3. How do you feel the county council worked to create legitimacy outside the organization for the cuts that needed to be made? In what way were citizens and patients involved in the development of the decommissioning programme? Were there any groups that you felt mobilized against planned cuts? What kind of cuts did they mobilize against?
4. How did the county council deal with people or groups who opposed the proposals in the decommissioning programme?

### The work of change: the distribution of power

1. What would you say that the distribution of power looked like between politicians, civil servants and the medical profession when the decommissioning programme was established? Did they have different perspectives on the problem definition and on solutions?

### Change management: the role of the media

1. What role do you think the media had in the preparation and adoption of the decommissioning programme? What role do you think the media has had in the subsequent period when the decisions are to be implemented?

### Change management: opportunity

1. Why would you say it was possible to make this decision (on the decommissioning programme), even though downsizing is often unpopular?

### Change management: implementation of decisions

1. How do you think the improvement work has been led and communicated?
2. At present: what are the most concrete efforts you are making to implement the decommissioning decisions that were made? What are the most concrete initiatives you feel that Dalarna County council is working on to implement the decommissioning programme?
3. Which actors are currently most important in implementing the decommissioning programme?
4. How do politicians and civil servants work to ensure that the decommissioning decisions adopted are also implemented?
5. In what way is Dalarna County council working to prevent similar problems from arising again? What are the longer-term changes you have implemented/or are working on?
6. How do you think the implementation of the decommissioning programme has worked (in general)?

### Closing questions

1. Is there anything else that you thought about before or during the interview?
2. Is there anything I haven't asked about that you wish to add?

**Source(s):** Authors' work.
